# Nonpharmacological interventions for the prevention of hypertension in low- and middle-income countries: a systematic review and meta-analysis

**DOI:** 10.1038/s41371-019-0223-x

**Published:** 2019-08-20

**Authors:** K. M. Saif-Ur-Rahman, Syed Shariful Islam, Md Hasan, Shahed Hossain, Razib Mamun, Sohana Shafique, Al Mamun, Md. Khalequzzaman, Fariha Haseen, Aminur Rahman, Iqbal Anwar

**Affiliations:** 1 Health Systems and Population Studies Division, icddr,b, Dhaka, Bangladesh; 2 Systematic Review Centre (SRC), Bangabandhu Sheikh Mujib Medical University (BSMMU), Dhaka, Bangladesh; 3 Department of Public Health and Informatics, Bangabandhu Sheikh Mujib Medical University (BSMMU), Dhaka, Bangladesh; 4 James P. Grant School of Public Health, BRAC University, Dhaka, Bangladesh

**Keywords:** Hypertension, Lifestyle modification

## Abstract

Hypertension is the single biggest cause of various cardiovascular complications and at the same time one of the most preventable phenomena. Low- and middle-income countries (LMICs) are facing increasing prevalence of hypertension which is imposing a huge burden on morbidity, premature mortality, and catastrophic health expenditure. This systematic review searched for the nonpharmacological interventions for prevention of hypertension among normotensive people in LMICs considering the period 1990–2016. This review has been conducted following standard methodology of Cochrane review involving two independent reviewers in screening, quality appraisal, and data extraction. Narrative synthesis of included articles was demonstrated using tables and meta-analysis was conducted to pool the estimates of studies which fulfilled the criteria. Total seven trials were included in the review with 6046 participants from eight LMICs. Two cluster randomized trials were pooled and there was a statistically significant effect (Systolic Blood Pressure: mean difference −2.35 [95% CI: −4.31 to −0.38], Diastolic Blood Pressure: mean difference −2.11 [95% CI: −3.20 to −1.02]) of home based health education in reducing blood pressure. Three individual studies reported reduction of blood pressure as a result of restricted dietary sodium intake. None of the studies was appraised as low risk of bias due to poor methodological quality. Non-pharmacological interventions can play important role in preventing the development of hypertension among normotensive people. Further trials with longer follow-up period and robust methods are recommended for getting stronger evidence on these interventions.

## Introduction

Hypertension attributes to the 6% of global burden of diseases and is responsible for 7.7 million global premature deaths annually [1]. Hypertension and its complications alone causes 53% cardiovascular diseases related mortality which is in the long run responsible for almost one third of total annual deaths [2]. Due to the epidemiological transition from infectious diseases to noncommunicable diseases (NCDs) and unhealthy sedentary lifestyle, low- and middle-income countries (LMICs) are experiencing the increasing trend of hypertension prevalence [3]. A systematic review and meta-analysis estimated that the overall prevalence of hypertension in LMICs is 32.3% [4]. This phenomenon is contributing to the dual burden of both infectious diseases and NCDs in LMICs and at the same time imposing the catastrophic healthcare expenditure to the communities and the nations [5].

Despite highly prevalent, hypertension is one of the most preventable conditions [6, 7]. Various studies have demonstrated different lifestyle modification approaches to reduce or prevent hypertension. Recommended measures for preventing hypertension are reducing body weight in case of obesity, undertaking regular physical activity, reduced intake of salt or sodium, increasing potassium supplement, and avoiding harmful use of alcohol [8]. Dietary intervention such as dietary approaches to stop hypertension has also promisingly reduced blood pressure (BP) [9]. This specific dietary recommendation included more intake of vegetables and fruits, milk products with lower proportion of fat, reduction of cholesterol, and saturated fat in meals [9]. A systematic review synthesizing information from 16 intervention studies including >3000 participants reported the reduction of both systolic and diastolic BP with increased calcium intake. Reduction of BP was even higher with increasing dose of calcium. Effect of calcium in reducing BP was greater among the younger population [10]. Complementary and alternative medicine has been found useful sometimes in this aspect [11–13]. A systematic review incorporated six RCTs and explored the effectiveness of “Yoga” either alone or in combination with conventional therapies in lowering BP. Results from this review also positively associated in reduction of BP although the quality of the included trials was notified as low grade [11]. The mind body therapy which is a combination of physical exercise and meditation was examined by one review that included nine RCTs, 13 quasi experimental studies and 4 observational studies. Pooled estimation depicted the significant effect of “Tai Chi”—a martial art originating in ancient China, embracing the mind, body, and spirit—in reducing BP. Only few of the included RCTs were methodologically strong in this systematic review [12]. An overview of systematic review investigated the effect of transcendental meditation—a technique for reducing stress on BP. A total of eight systematic reviews including Cochrane reviews were included. Overall the assessment was fare in terms of the quality of the included reviews. Results from the overview also supported the role of meditation in lowering BP despite some conflicting results between included reviews [13]. Apart from dietary modifications, changing lifestyle, alternative medicine and meditation, therapeutic agent such as combination of Chlorthalidone and Amiloride have also been tested. This double blinded, placebo controlled randomized trial demonstrates the significance of the therapeutic agent in preventing hypertension [14]. Despite significant effect on prevention, there is risk of experiencing adverse effect among the participants. Taking regular medication for prevention may also raise question on compliance [15].

In addition, most of these interventions are based on high-income countries. LMICs are challenged with limited resources to provide useful programs for the early diagnosis, prevention, or control of this huge burden of disease [2, 7, 16]. Interventions for prevention of hypertension among persons with normal BP (Systolic BP (SBP) 120–139 mmHg and diastolic BP (DBP) 80–89 mmHg) [17] or prehypertensive (SBP ≥ 120–139 mmHg and/or DBP ≥ 80–89 mmHg) [18] can play an important role to tackle this progressively increasing disease and reduce complications and morbidity resulting from that. Our objective of the present systematic review is to explore all available interventions which are nonpharmacological in approach and to synthesize their effectiveness in prevention of hypertension in LMICs.

## Methods

This systematic review has been carried out following the methodology of Cochrane systematic reviews [19] and addressed the requirements stated in preferred reporting items for systematic reviews and meta-analysis protocols guidelines [20, 21]. Details methodology including the development of search strategy, dual-screening process, dual-data extraction, dual appraisal of included articles for quality assessment, narrative synthesis, and meta-analysis has been described in the published protocol [22]. A comprehensive search strategy was developed using the key words such as Exercise, “Physical activity”, “Weight loss”, “Sodium restriction”, “Dietary potassium”, “Calcium supplementation”, “Fish oil supplementation”, Lifestyle, Hypertension, “Blood pressure” to search different electronic bibliographic database including Embase, MEDLINE through Pubmed, Web of Science, Clinical Trials. gov., the Cochrane Library (Cochrane Central Register of Controlled Trials (CENTRAL), Scopus etc. The search period covered from 1990 to 2016. Randomized control trials providing nonpharmacological intervention on normotensive adult population in LMICs were included. Both the screening of “title and abstract” and “full text” of the retrieved articles were conducted independently by two reviewers and any disagreement was resolved by a third reviewer. Reference management software was used to keep track of the screening process. Each of the studies was appraised critically for assessment of risk of bias (ROB). A narrative synthesis of the characteristics of study participants and types of intervention with specific outcome was demonstrated. Mean and standard deviation of both systolic and diastolic BP were recorded from baseline and endline information. For meta-analysis, a random effect model was chosen with 95% confidence interval and both the chi squared and I^2^ statistic were measured. The systematic review is registered in International Prospective Register of Systematic Reviews. Registration number is CRD42017055423.

## Results

**Fig. 1:**
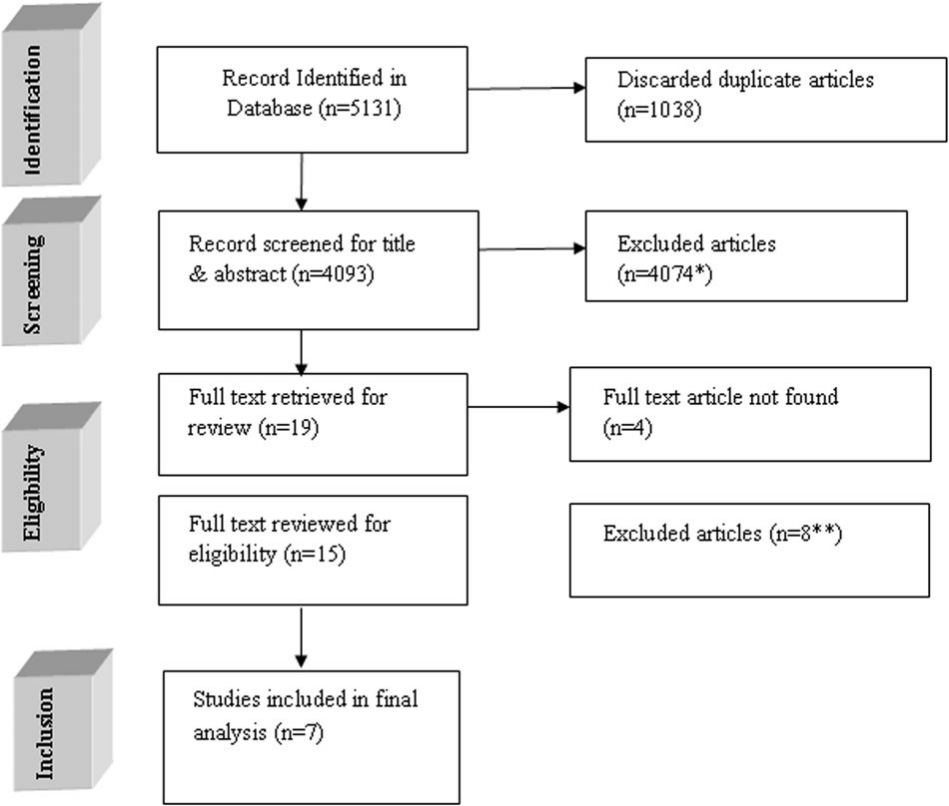
Flow diagram of the inclusion process

A total of 5131 articles were retrieved after searching eight selected database using a comprehensive search strategy. After removing the duplicates, 4093 articles were compiled for title and abstract review. Applying inclusion and exclusion criteria, 19 articles were selected for full text review. We did not found the full text of four articles even after communicating with the corresponding author. The main causes of excluding these articles were irrelevance with the review objective. Out of these 15 articles, eight articles were excluded due to the following reasons: four articles were not focusing LMIC’s; two articles did not follow RCT design; one article was a literature review and intervention was given on hypertensive population in one article. After screening, seven RCTs were included in the final analysis. The detailed description of the selection process of the included articles has been provided in Fig. 1 using PRISMA flow diagram.

### Quality assessment of the included studies

**Fig. 2:**
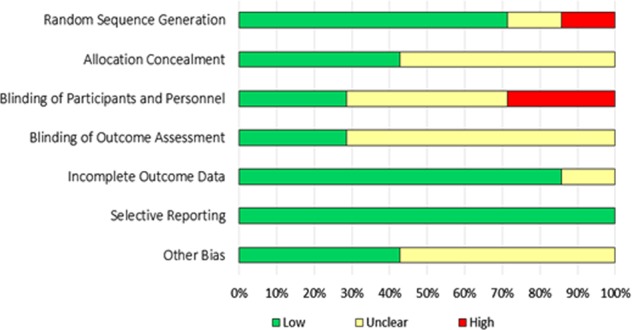
Overall risk of bias among the included articles

**Fig. 3:**
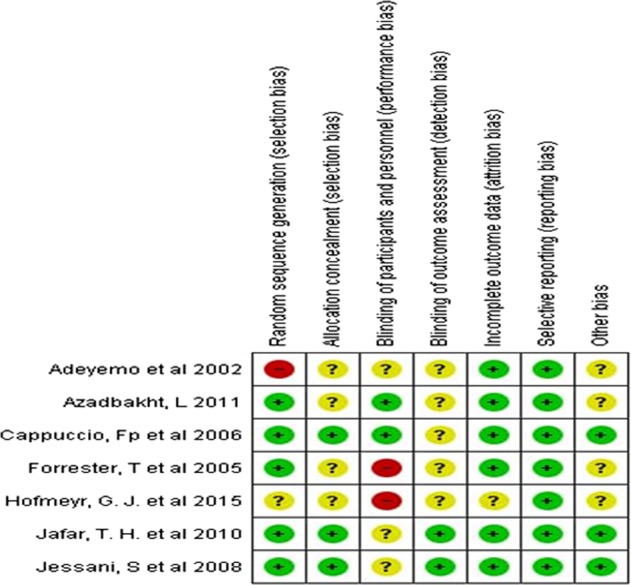
Risk of bias assessment in individual studies

We assessed the ROB of the included trials using the guideline of Cochrane review. Majority of the trials (five out of seven) performed the sequence generation randomly and reported accordingly. Only three articles described the process of allocation concealment. Thus, there was potential risk of selection bias in almost half of the trials. Only two studies maintained blinding at the level of participants and implementers. Another two trials mentioned about blinding at the level of outcome assessors. Overall, majority (five in each case) of the trials were unable to minimize the chance of performance bias and detection bias. Almost all the studies mentioned about attrition rate. Only one study did not describe regarding attrition and marked as unclear information. All the articles were with sufficient information regarding the primary outcome hence we recognized all of them as at low risk for selective reporting bias. Majority of the studies did not provide any information regarding other potential biases. We did not get the description regarding contamination in cluster randomized trials and rationale behind the duration of washout period in the studies with crossover design. A graphical demonstration of assessment of ROB has been provided in Figs. 2, 3.

A summary description of the basic characteristics of the included articles has been given in Table 1. Among the seven included RCTs, two studies used clustered randomized design [23, 24], two studies were randomized control trials [25, 26] and three studies were with crossover design [27–29]. Among the three crossover trials, two studies incorporated a washout period of 1 and 3 weeks, respectively [28, 29] before altering the treatment options. All the studies described the effectiveness of the intervention to prevent hypertension. The main outcome, BP, was measured manually with two exceptions which considered both manual and electronic measurements [25, 28]. The interventions provided by different trials were health education program, soy drink and cow’s milk; calcium tablets; and low- or high-salt intake. Among these studies only two met the criteria of meta-analysis and rest of the studies were described using summary statistics. All the included studies considered both male and female except one [26] where intervention was provided among the nonpregnant women. Sample size of the studies varied based on the design adapted. Cluster randomized trials included large number of participants [23, 24] whereas some RCTs included <100 participants [25, 27]. Four studies were conducted in African region [23, 25, 26, 28]; one was in Middle East [27] and two studies were conducted in South Asia [24, 29]. All the studies included adult respondents and only one study included participants aged between 5 and 39 years [24].

**Table 1:** Characteristics of the of the included articles

Author	Year of Publication	Sample Size	Study design	Country	Age in Year	Gender	Intervention	Duration of intervention
Cappuccio et al. [1]	2006	I—522 C—491	RCT—cluster randomized design	Ghana	I-54 C−55	M and F	Health education vs control	Daily meeting for the first week—once weekly thereafter.
Azadbakht et al. [2]	2011	I and C—23	RCT—crossover design	Iran	18–30	F	Soy drink vs cow’s milk	6 weeks
Adeyemo et al. [3]	2002	82	RCT	Nigeria	>25	M and F	Reduced salt intake	2 weeks
Hofmeyr et al. [4]	2015	I—1st trial; 1st—181, 2nd—97 C—2nd trial; 1st—186, 2nd—104	RCT	South Africa, Zimbabwe and Argentina	≥18, mean 30.3	F	Calcium tablet	12 weeks (1st trial visit) 24 weeks (2nd trial visit)
Forrester et al. [5]	2005	Nigeria 58, Jamaica 56	RCT—crossover design	Nigeria, Jamaica	25–55	M and F	Low-salt vs high-salt diet	8 weeks (intervention 3 weeks, follow up 2 weeks, washout 3 weeks)
Jessani et al. [6]	2008	200	RCT—crossover design	Pakistan	≥40	M and F	Low sodium vs high sodium	3 weeks (intervention 1 week, follow up1 week, washout 1 week)
Jafar et al. [7]	2010	I 2008 C 2015	RCT—cluster randomized design	Pakistan	May-39	M and F	Health education vs control	3 months

*I* Intervention group, *C* control group, *M* Male, *F* Female

Table 2 showed the results of studies that were not included in meta-analysis. These studies were heterogeneous enough for not including in the meta-analysis in terms of interventions, duration of the study, and study settings. Azadbakht et al. measured the beneficial effect of soy milk in comparison with cow’s milk on anthropometric measurements and BP for overweight and obese female youths [27]. It showed that the SBP was reduced significantly following the soy drink intervention period than that of the cow’s milk. Mean percent change during soy milk period was −0.4 ± 0.9 and −1.7 ± 0.5 during cow’s milk period. Diastolic BP was also reduced in soy milk drink period (−0.4 ± 0.1 vs 0.4 ± 0.1). Adeyemo et al. provided dietary intervention among normotensive adults in South East Nigeria to determine the feasibility of reducing dietary sodium intake [25]. BP of the participants was measured using both manual procedure and electronic device. In manual measurement, among the participants on low sodium diet, SBP was reduced by −4.7 ± 2.8 mmHg for men and by −7 ± 4.4 mmHg for women. The diastolic BP was reduced by −1.9 ± 2.2 mmHg among men and by −1.6 ± 3.4 mmHg among women. Hofmeyr et al. explored the effect of calcium on the BP among nonpregnant women who experienced pre-eclampsia previously [26]. The follow-up was conducted in two phases at 12 weeks and 24 weeks, respectively. Participants were provided 500 mg calcium tablet each day. Overall, the BP in calcium supplementation group was reduced but that was not statistically significant. Forrester et al. compared the effectiveness of high-salt diet (usual diet and additional 50 mEq salt pills) and low-salt diet (usual diet and a reduction of 50 mEq salt) on BP [28]. This study was conducted in two regions of Nigeria and Jamaica and results were compared between these two zones as well. BP was measured using both electronic automated machine and manual procedure. The average result from the two procedures was used in the final analysis. The mean change in SBP between low and high sodium diet phase was ~5 mmHg in both groups. This study depicted that there is a significant efficacy of sodium reduction in lowering the BP. These results were consistent with the studies conducted among affluent population in high income countries. Jessani et al. also estimated the effect of high and low sodium diet among the Pakistani population [29]. Participants were randomly allocated either in a low sodium diet group (20 mEq/day) or a high sodium diet group (220 mEq/day) for 1 week. The washout period for both the groups was 1 week before the crossover phase. The crossover period with the altered diet was for another 1 week. Researchers measured the difference in SBP and diastolic BP in each phase as primary outcome. SBP was classified as high normal SBP and normal SBP. The result showed that adjusted mean of SBP reduced significantly among the participant with high normal SBP (130–139 mmHg) during low sodium diet in comparison with baseline. There were no significant changes among the participants with normal SBP (<130 mmHg) at the baseline.

**Table 2:** Summary results of the studies not included in meta-analysis

Authors	Intervention	SBP				DBP				Mean percent changes			
		Baseline/placebo		Endline/intervention		Baseline/placebo		Endline/intervention		SBP		DBP	
		Mean	SD	Mean	SD	Mean	SD	Mean	SD	Mean	SE	Mean	SE
Azadbakht et al. [2]													
	Cow’s milk	102.1	2.2	100	2.1	65.6	2.2	65.8	2	−1.7	0.5	0.4	0.1
	Soy milk	100.8	2.1	96	2.2	66.3	2	66	2.2	−4	0.9	−0.4	0.1
Adeyemo et al. [3]	Manual Low vs high sodium diet—women	110.1	14.6	103.1	9.8	69.1	11.5	67.5	7.5	−7	4.4	−1.6	3.4
Adeyemo et al. [3]	Low vs high sodium diet—men	116.8	15.3	112.1	12.5	74	9.5	72.1	9.5	−4.7	2.8	−1.9	2.2
Hofmeyr et al. [4]	1st trial—Placebo vs Calcium supplementation	126.1	16.3	127.4	17.2	81.6	11.5	81.5	13	1.4	3	1	2.4
	2nd trial—Placebo vs Calcium supplementation	126.8	16	131	19.6	81.9	11.4	83.6	14.5	2.5	4.4	1.4	3.5
Forrester et al. [5]	Nigeria—high vs low sodium phase	114.8	11.4			73.3	9.1			4.5	2.9	2.7	2
	Jamaica—high vs low sodium phase	122.3	10.2			75.9	7.3			5.5	1.5	2.8	2.3
Jessani et al. [6]	High sodium phase	122	11	134	40	79	6	82	13.5	6	3	0	1
High normal SBP	Low sodium phase	122	11	128	20	79	6	81	13.5				
Jessani et al. [6]	High sodium phase	122	11	118	27	79	6	82	13.5	−1	1	0	1
Normal SBP	Low sodium phase	122	11	119	27	79	6	81	13.5				

### Meta-analysis

**Fig. 4:**
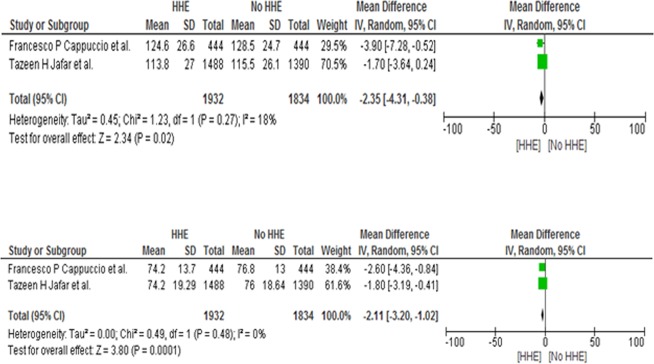
Comparison between home health education (HHE) and no HHE, outcome effect mean difference of systolic blood pressure and diastolic blood pressure

In this analysis, only two articles fulfilled the criteria of meta-analysis. Pooled estimate revealed a significant reduction in BP of home based health education with control (pooled mean difference SBP: −2.35 [95% CI −4.31, −0.38] mmHg, *p* = 0.02; pooled mean difference DBP: −2.11 [95% CI −3.20, −1.02] mmHg, *p* = 0.0001) (Fig. 4). Heterogeneity between these cluster randomized trials was very low (SBP: *I*^2^ = 18%; DBP: *I*^2^ = 0%) which implies that there is necessity to conduct meta-analysis without subgroup analysis. The forest plot is demonstrated in Fig. 4.

### Publication bias

In our review, it was not possible to observe the publication bias because of very few numbers of included studies for meta-analysis. Funnel plot generally used to estimate the risk of publication bias. For only two studies, result of this graph is unpredictable. It is also recommended that test of funnel plot asymmetry or existence of publication bias is not possible if the selected study is <10 in meta-analysis [30].

## Discussion

With an aim to examine the effectiveness of nonpharmacological interventions for prevention of hypertension in LMICs, this systematic review included seven trials incorporating 6046 patients from eight countries over the last 16 years. Although a comprehensive search was undertaken, just seven studies met the inclusion criteria. The studies investigated a range of interventions on normotensive population including health education, soy drink, calcium supplementation, and low sodium diet. Only of two studies were eligible to [23, 24] combine the outcomes through meta-analysis which showed the effects of health education on reducing BP in normotensive participants. The significant change in BP indicates that positive effect of health education among normotensive individuals in reducing their BP. However, there were dissimilarities in number and age group among two study participants. Health education for the prevention of hypertension is widely used in the interventions which covered prehypertension individuals. Moreover, this approach is also used for normotensive individuals to make them aware. Study showed that community-based health education program on hypertension and cardiovascular risk factors resulted into declining BP of the participants in the intervention group [31]. The behavior change communication messages mentioned in the included articles in this review focused on knowledge of lifestyle modification such as lowering salt intake in food [23], increased physical activity, cessation of smoking, consumption of low fat diet including dairy products, and increased intake of fruits and vegetables [24]. Another systematic review demonstrated the effectiveness of lifestyle modification on metabolic syndrome where information from eight trials were pooled together [32].

In this review, three studies reported effect on reducing BP through reduction in dietary sodium intake [25, 28, 29]. Intervention period in these studies were short, ranging from 2 to 8 weeks. The result showed significant effect of sodium intake modification, it is worth mentioning that the three included studies varied in their findings, reporting different range of changes in SBP and DBP. A Cochrane review also demonstrated the significant change in BP among White, Black, and Asian people with normal BP where there was a greater reduction rate among Black and Asian people [33].

One RCT (crossover design) focused on the effect of soy drinks and cow milk among the females [27]. SBP reduced significantly but no significant changes in DBP due to the intervention of soy drink. However, included participants were obese and on a weight reducing diet. Calcium tablet was given to intervention group in one study [26] where no significant change in reducing BP was observed. Similar results has been demonstrated in a systematic review incorporating sixteen trials [10] where slight reduction of BP was observed due to increase in dietary calcium intake. However, these trials could not conclude with strong recommendations for this specific intervention.

As per world health organization, sodium reduction in food intake can reduce BP in normotensive individuals [2]. In this systematic review, a trend toward reduction in BP was observed by dietary sodium reduction. Studies conducted by Law et al. and Miller et al. [34, 35] reveled that participants having higher BP at baseline resulted in greater reductions in BP due to the effect of sodium reduction. Many studies demonstrated that decreasing sodium in diets have benefits in lowering BP among prehypertensive participants [36–38]. However, one study showed that the reduction of dietary sodium has no significant effect in normotensive individuals with SBP < 130 mmHg and with normal kidney function [39]. Another crossover study reported no significant changes in BP due to the reduction of salt intake [40].

No studies on physical activities related intervention to reduce hypertension was found in LMICs. However, meta-analysis of seven studies conducted among Brazilian population also demonstrated the significant impact of resistance exercise and aerobics in lowering both SBP and DBP [41]. Pooled estimate showed reduction in both systolic and diastolic pressures which was statistically significant. But the sample size of included studies were very small with short intervention period and both normotensive and hypertensive patients were included. Another systematic review and meta-analysis conducted demonstrated that isometric handgrip exercise is efficacious for reducing SBP and DBP in adult participants [42].

We conducted a broad search of several databases but placed restrictions on the language of the study when searching the electronic databases. Studies published in english language were only considered in this review which is one of the main limitations. It is likely that there are other studies published in other languages which we have missed in this review. Strengths of this systematic review are the inclusion of RCTs only and following Cochrane guideline strictly.

None of the included studies was found as low ROB and also methodologically none of the studies was of high quality. In some studies, short duration of intervention period with small sample demonstrated quick significant results but their sustainability remained questionable. Follow-up after a longer duration could have observed for the sustainability of the impact of interventions. Studies did not mention about potential confounders and contamination in case of cluster randomized trials.

## Conclusion

This review demonstrated the effectiveness of nonpharmacological interventions for prevention of hypertension of the included trials. Given the limited scientific evidence in LMICs and quality of the evidences, no strong conclusion about effectiveness of nonpharmacological approaches could be drawn. This systematic review highlights the need of future research opportunities and a necessity for more scientific studies with larger numbers of participants and longer intervention period using robust study design.

### Summary

#### What is known about topic

- Hypertension attributes to the cardiovascular disease burden and responsible for premature death.
- Different non pharmacological approaches have been implemented for prevention of hypertension in high income countries

#### What this study adds

- Explored all available non pharmacological intervention trials in LMICs
- Home based health education has got significant effect in preventing hypertension among people with normal blood pressure in LMICs
- More researches with robust methodology and longer follow up are required for recommending other non pharmacological approaches

## Data Availability

The datasets generated and/or analyzed during in this review shall be available from the corresponding author on reasonable request.
